# Primary choroidal lymphoma with extrascleral extension and bone marrow involvement: a case report

**DOI:** 10.3389/fmed.2025.1638453

**Published:** 2025-10-27

**Authors:** Lu Zhao, Yingyu Li, Pei Zhang, Changguan Wang, Chunyuan Li, Huijin Chen

**Affiliations:** 1 Beijing Key Laboratory of Restoration of Damaged Ocular Nerve, Department of Ophthalmology, Peking University Third Hospital, Beijing, China; 2 Beijing Ophthalmic and Visual Science Key Laboratory, Beijing Tongren Eye Center, Beijing Tongren Hospital, Capital Medical University, Beijing, China; 3 Department of Hematology, Lymphoma Center, Peking University Third Hospital, Beijing, China

**Keywords:** extranodal marginal zone B-cell lymphoma, choroid, lymphoma, bone marrow, involvement

## Abstract

Primary extranodal marginal zone B-cell lymphoma (EMZL) of the choroid is a rare condition with a handful of cases reported in the literature. Here, we report a challenging case of primary EMZL of the choroid with extrascleral extension and bone marrow involvement. An 86-year-old male presented with 2 years of progressive vision loss in his left eye. Fundus examination revealed yellow-white subretinal infiltrates at the posterior pole. Ultrasonography detected diffuse choroidal thickening with retrobulbar hypoechoic mass. One year later, an inferior exudative retinal detachment developed. Ultrasonography revealed enlargement of the choroidal low-reflective mass. Another half a year later, visual acuity of the left eye deteriorated to no light perception (NLP) and neovascular glaucoma with intractable pain necessitated enucleation; therefore, enucleation was performed. Histopathological and immunohistochemistry findings confirmed EMZL infiltrates in the choroid and extrascleral tissue, without optic nerve involvement. Concurrent hematologic evaluation revealed significant anemia. The patient was transferred to the hematology department. Bone marrow biopsy demonstrated EMZL involvement morphologically and immunophenotypically identical to the choroidal tumor. Positron emission tomography-computed tomography (PET-CT) revealed no evidence of metabolically active lesions in the lymph nodes, spleen, or visceral organs to suggest an alternate primary tumor. Thus, a diagnosis of primary choroidal EMZL with extrascleral extension and bone marrow involvement was established. To our knowledge, this represents the first reported case of primary choroidal EMZL with bone marrow involvement.

## Introduction

Intraocular lymphoma is anatomically divided into vitreoretinal lymphoma and uveal lymphoma. Most intraocular lymphomas are vitreoretinal lymphoma, while uveal lymphoma is rare. Vitreoretinal lymphoma is highly aggressive, with 60–85% of patients developing central nervous system lymphoma (1). Approximately 95% of vitreoretinal lymphoma cases are diffuse large B-cell lymphomas (DLBCL), while T-cell and natural killer/T-cell lymphomas are uncommon (2). In contrast, uveal lymphomas are typically low-grade and follow an indolent clinical course; aggressive cases are rare (2). Within the uvea, the choroid is the most frequent site of involvement, whereas the iris and ciliary body are rarely affected (3). Choroidal lymphoma can be further classified as primary or secondary. The predominant pathological subtype of primary choroidal lymphoma is extranodal marginal zone B-cell lymphoma (EMZL). Primary choroidal EMZL typically presents as a localized uveal mass that may exhibit extrascleral extension; this subtype is indolent, carries a good prognosis, and rarely involves the central nervous system or bone marrow (4, 5). Historically, many choroidal EMZL cases were diagnosed only after enucleation, performed either due to diagnostic challenges in distinguishing lymphoproliferative lesions from malignant uveal melanoma, or to manage pain secondary to glaucoma. Here, we present a notably rare case of primary choroidal EMZL with extrascleral extension and bone marrow involvement. Written informed consent was obtained from the patient, and this study adheres to the tenets of the Declaration of Helsinki.

## Case presentation

**Figure 1:**
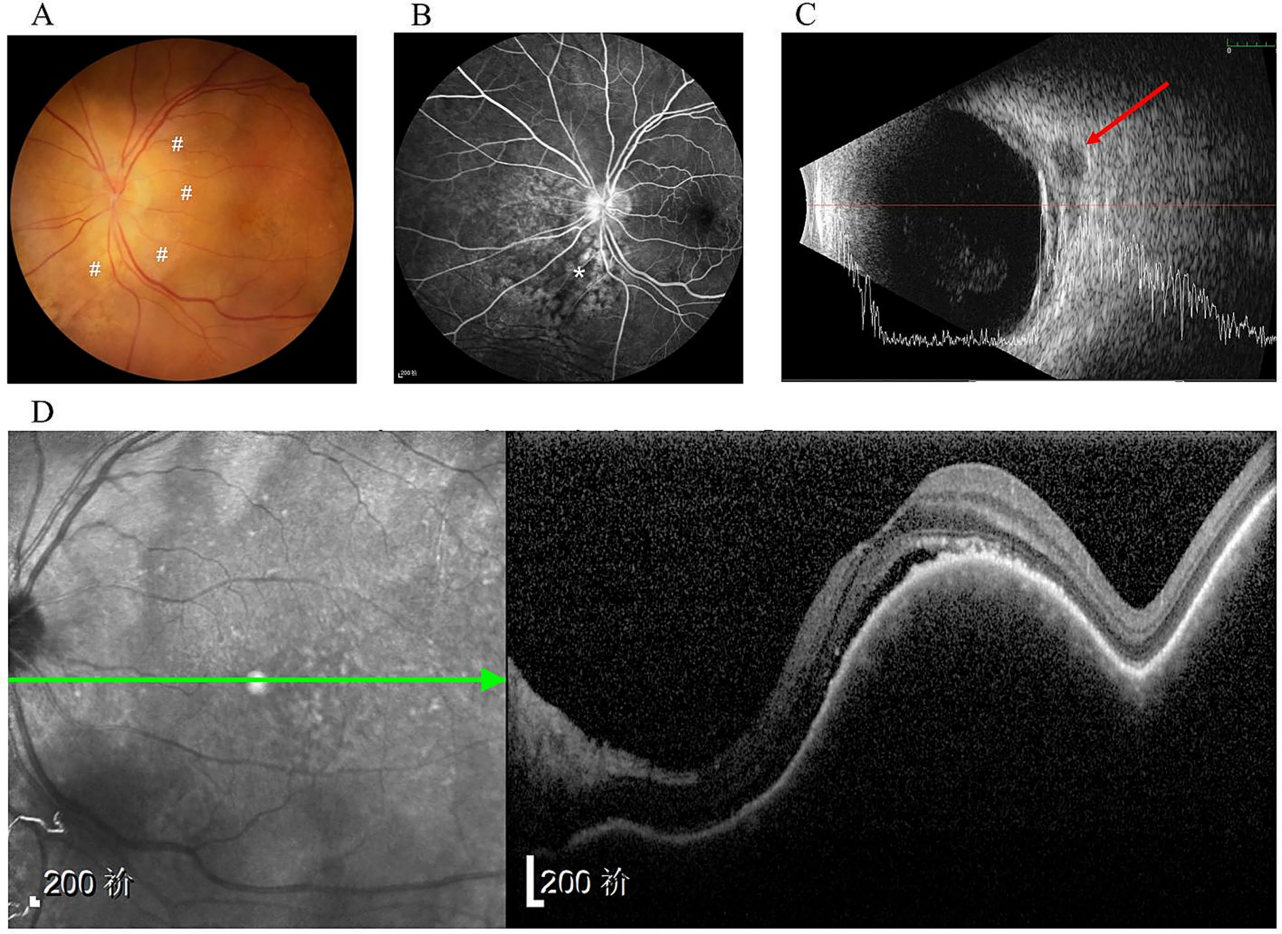
Initial presentation. **(A)** Fundus examination showed an elevated yellow-white sub-retinal infiltrates (#); **(B)** FFA taken at 3 min showed mottled hyper-fluorescence area (*); **(C)** Ultra-sonography showed diffuse choroidal thickening and retrobulbar hypoechoic nodule (arrow) in the left eye. **(D)** OCT of the left eye showed domed choroid thickening on the macula.

An 86-year-old male was admitted to the Department of Ophthalmology at the Peking University Third Hospital in May 2021 for the first time. He presented with 2-year progressive vision loss in his left eye. The patient had no significant medical or family history except bilateral pseudophakia following prior cataract surgery. On initial examination, best-corrected visual acuity (BCVA) was 20/40 in the left eye, with normal intraocular pressure (IOP). The examination of right eye was unremarkable. Anterior segment and vitreous examination of the left eye appeared normal. However, fundoscopy revealed an elevated yellow-white subretinal lesion at the posterior pole (Figure 1A). Fluorescein angiogram (FA) showed mottled hyperfluorescence in the choroid (Figure 1B). Ultrasonography demonstrated diffuse choroidal thickening and retrobulbar hypoechoic mass (Figure 1C). Optical coherence tomography (OCT) showed dome-shaped choroid thickening and retinal pigment epithelium (RPE) changes under the macula (Figure 1D). Chest computed tomography (CT) scans indicated a 12 × 27 mm nodule in the right lung. Concurrent hematologic tests revealed anemia (hemoglobin level 88 g/L). Workup showed a high reticulocyte index, unremarkable lactate dehydrogenase (LDH) and haptoglobin levels, and normal levels of iron, vitamin B12, and folate, consistent with anemia of chronic disease. No other cytopenias were present. Given concern for metastatic disease, positron emission tomography-computed tomography (PET-CT) along with needle biopsy of the pulmonary nodule and bone marrow aspiration were recommended; however, the patient declined these procedures. Consequently, no treatment was initiated for the left eye pending definitive diagnosis.

**Figure 2:**
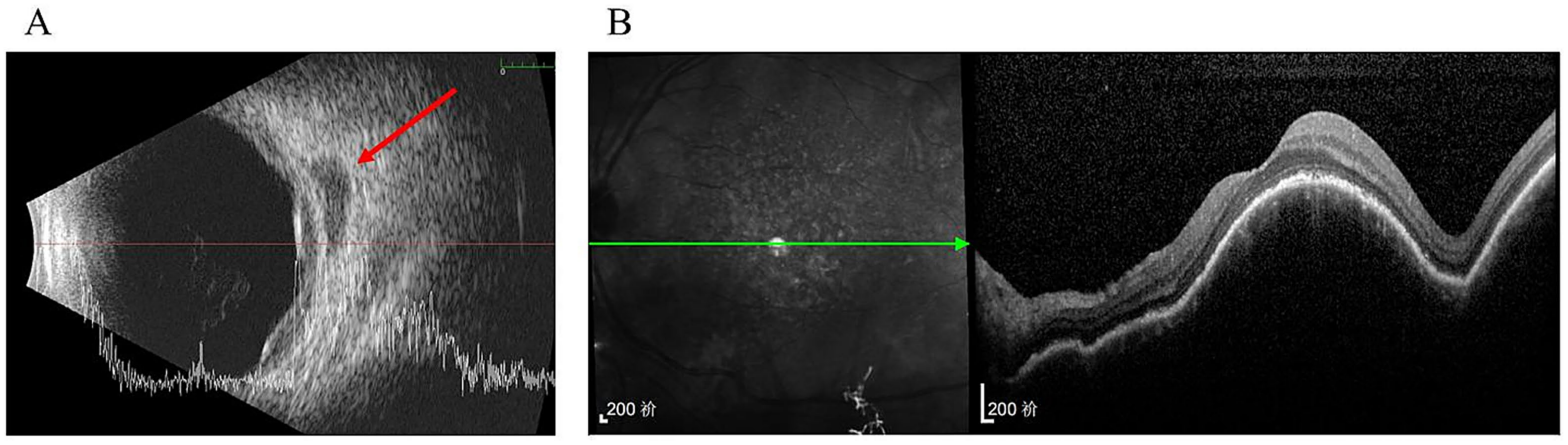
Three months after the initial presentation. **(A)** Ultrasonography showed the area of diffuse choroidal thickening and retrobulbar hypoechoic area were slightly increased (arrow); **(B)** No significant change in the performance of OCT compared with the presence of last time.

Three months later, ultrasonography demonstrated only slight enlargement of the choroidal thickening region and hypoechoic area (Figure 2A). Furthermore, OCT imaging at the macula showed no significant changes compared with the previous scan (Figure 2B).

**Figure 3:**
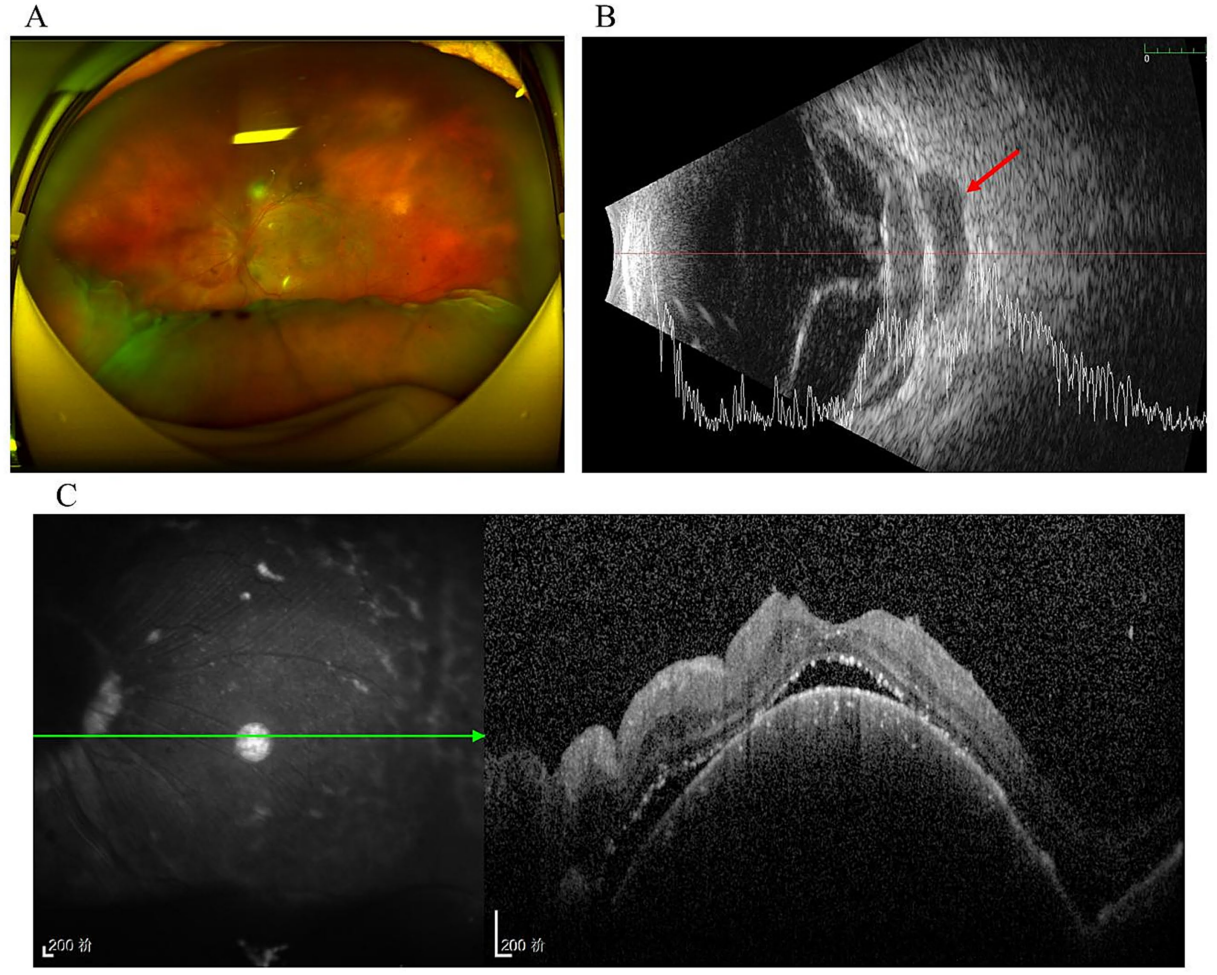
One year after initial presentation. **(A)** Fundoscopy showed inferior exudative retinal detachment in the left eye; **(B)** Ultrasonography revealed that retinal detachment, the area of diffuse choroidal low-reflective mass and retrobulbar hypoechoic were significantly enlarged (arrow); **(C)** OCT of the left eye showed domed choroid thickening and retinal folds.

**Figure 4:**
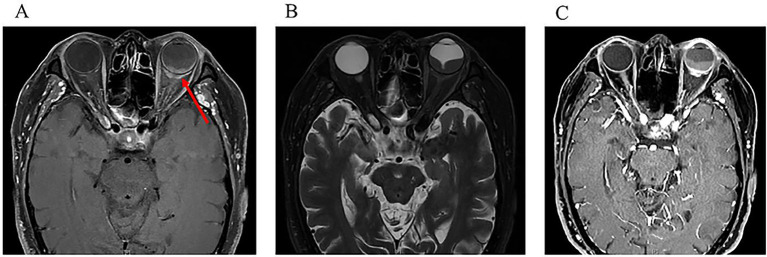
One year after initial presentation. An MRI scan of the orbits showed a mass (arrow) of irregular shape, predomi-nantly affecting the posterior parts of the eyeball, as well as transscleral extension. **(A)** Horizontal view of T1-weighted sequences; **(B)** Horizontal view of T2-weighted se-quences; **(C)** Horizontal view on contrast-enhanced in T1-weighted sequences.

The patient was lost to follow up for 1 year and returned to our department in August 2022 due to the deterioration of visual acuity in his left eye. Fundoscopy revealed an inferior exudative retinal detachment in the left eye (Figure 3A). Ultrasonography showed significant enlargement of the diffuse choroidal low-reflectivity mass and retrobulbar hypoechoic areas (Figure 3B). Furthermore, OCT of the left eye showed dome-shaped choroidal thickening with retinal folds (Figure 3C). Magnetic resonance imaging (MRI) of the head and orbits demonstrated extensive choroidal thickening with a maximal height of 2.8 mm, along with fusiform soft tissue encasing the optic nerve that appeared isointense on both T1- and T2-weighted sequences and exhibited mild heterogeneous enhancement; notably, there was no evidence of adnexal or central nervous system (CNS) involvement (Figure 4). Tumor markers and immunohistochemistry tests yielded normal results, excluding malignancies and autoimmune diseases. Consequently, the patient was referred to respiratory and hematology departments for a visit. Repeat chest CT showed stability of the pulmonary nodule compared with the prior scan. Although lung biopsy was recommended by pulmonologist, the patient declined the procedure.

Another half a year later, on January 2023, the patient’s left eye visual acuity deteriorated to no light perception (NLP) with elevated intraocular pressure (50 mmHg, 1 mmHg = 0.133 kPa). Ophthalmoscopic fundus visualization was obscured by hyphema secondary to neovascular glaucoma. Ultrasonography confirmed vitreous hemorrhage and total retinal detachment, with significant enlargement of the choroidal thickening and retrobulbar hypoechoic area compared to the previous examination (Supplementary Figure 1A). Given NLP vision, enucleation was performed after obtaining written informed consent from the patient. There was a diffused elevated yellow-white mass in the posterior surface of the eyewall, encircling the optic nerve (Supplementary Figure 1B). Histopathological and immunohistochemistry examination findings confirmed extranodal marginal zone lymphoma involving the choroid with massive extrascleral extension (Supplementary Figures 2A–C). Furthermore, immunohistochemical results showed that neoplastic cells were positive for CD20, with a low proliferation index of <5% determined by Ki67 labeling. Notably, the iris, ciliary body, and optic nerve (Supplementary Figure 2D) were not involved. PET-CT showed increased metabolic activity in proximal/axial bones, consistent with lymphoma infiltration, but no abnormalities in the lungs, lymph nodes, spleen, or visceral organs. The patient was then transferred to the hematology department for further systemic management. Bone marrow aspiration examination revealed active proliferation of nucleated cells, indicating the total lymphoid cells accounted for 66.5%, with immature lymphocytes comprising 2.5% of this population (Supplementary Figure 2E). Flow cytometry detected 45.72% abnormal mature B-cells (CD5^−^/CD10^−^), excluding chronic lymphocytic leukemia/small lymphocytic lymphoma and mantle cell lymphoma while supporting marginal zone lymphoma. Bone marrow biopsy demonstrated EMZL infiltration morphologically and immunophenotypically identical to the choroidal tumor. Supportive care for anemia was provided, and a plan was established for regular clinical and laboratory follow-up, with rituximab therapy to be initiated upon evidence of disease progression. With over 4 years of follow-up since initial presentation, the patient survives with preserved quality of life.

## Discussion

To our knowledge, this represents the first reported case of primary choroidal marginal zone lymphoma with bone marrow involvement. The choroid is definitively established as the lymphoma origin through converging evidence including temporal precedence of visual deterioration over systemic anemic manifestations, histopathologic confirmation of extranodal marginal zone B-cell lymphoma exhibiting CD20^+^ and Ki67 < 5% without anterior segment involvement in the enucleated mass, PET-CT exclusion of alternate primaries showing no metabolically active lesions in lymph nodes, spleen, or visceral organs, and bone marrow biopsy revealing EMZL morphologically and immunophenotypically identical (CD5^−^/CD10^−^) to the choroidal tumor. This finding is further supported by flow cytometry detection of 45.72% aberrant CD5^−^/CD10^−^ mature B-cells, a result that excludes chronic lymphocytic leukemia/small lymphocytic lymphoma (CLL/SLL) and mantle cell lymphoma. Post-enucleation disease control further confirms primary choroidal EMZL with secondary hematogenous dissemination.

Orbital and ocular adnexa lymphomas (OOAL) are a rare localization of lymphomas confined to the orbital region, accounting for approximately 7–8% of all non-Hodgkin’s lymphomas (NHLs) (6, 7). These neoplasms develop from B-lymphocytes, T-lymphocytes, or NK lymphocytic cells (8). Within the NHLs, histological subtypes can be divided into indolent or low-grade lymphoma (i.e., EMZL) involving mucosa-associated lymphoid tissue (MALT), follicular lymphoma (FL) or lymphoplasmacytic lymphoma) and aggressive or high-grade lymphomas (e.g., diffuse large B-cell lymphoma (DLBCL) or mantle-cell lymphoma (MCL) with different patterns of response to local and systemic treatment, local or distant recurrence (9).

Intraocular lymphoma is relatively rare. The most common form is primary vitreoretinal lymphoma, which exhibits aggressive behavior and is predominantly DLBCL. In comparison, uveal lymphoma is less frequent. Among uveal cases, the choroid is the primary site of involvement, with the iris and ciliary body rarely affected (3). Choroidal lymphoma is classified as primary or secondary. Primary choroidal lymphoma typically presents as an indolent, low-grade tumor confined to the choroid, historically termed reactive lymphoid hyperplasia or uveal pseudotumor. Conversely, secondary choroidal lymphoma occurs in patients with advanced systemic lymphoma/leukemia (2, 10, 11), usually as a high-grade malignancy with rapid progression. These clinical differences correlate with distinct pathological subtypes. The predominant subtype in primary choroidal lymphoma is EMZL, whereas systemic lymphomas involving the choroid are most commonly DLBCL, with rare cases of multiple myeloma or lymphoplasmacytic lymphoma (2).

The exact incidence of primary choroidal lymphoma remains unknown due to limited reported cases. According to the Revised European-American Lymphoma (REAL) classification for EMZL, approximately 80 cases have been documented since 1994 (4, 12, 13). The largest choroidal lymphoma series from Wills Eye Hospital included 59 patients (73 eyes) (12), with none developing systemic disease during a 27-month follow-up. Aronow et al. (14) reported 22 choroidal lymphoma cases: choroidal involvement in 21 (95.5%) and concurrent ciliary body involvement in one (4.5%), with no systemic progression over a mean 28.7-month follow-up. Thus, this represents the first reported case of primary choroidal EMZL with bone marrow involvement, suggesting potential hematogenous dissemination of choroidal lymphoma. The mechanisms underlying the dissemination of choroidal lymphomas remain unclear. Building on previous studies, the involvement of MALT lymphoma to the bone marrow is identified as a complex process involving various biological mechanisms. We speculate that choroidal lymphomas spread to bone marrow through lymphatic and vascular systems (15). The interaction between lymphoma cells and their microenvironment plays a crucial role in their dissemination. Cytokines in tumor microenvironment such as tumor necrosis factor-*α*, interleukin (IL)-6, IL-10 and transforming growth factor-*β* may enhance this process, promoting tumor cell proliferation and survival, thereby affecting tumor spread to hematological system (16). In addition, genetic factors may also contribute to the spread of choroidal lymphoma, with certain genetic mutations potentially enhancing the lymphoma cells’ dissemination capability (17). This includes changes in gene expression related to cell migration and infiltration, suggesting that further genetic material testing is needed to provide robust evidence. Therefore, a thorough and careful ocular and systemic examination is of paramount importance in the management of any suspected patient. In a patient with choroidal lymphoma who suffered from anemia, bone marrow involvement require exclusion.

Due to the rarity of primary choroidal lymphoma, limited data exist on its characteristic diagnostic features. Moreover, the clinical manifestations of primary choroidal lymphoma are often insidious and nonspecific (18). Several ophthalmic conditions can mimic choroidal lymphoma, including amelanotic choroidal melanoma, posterior scleritis, choroidal metastases, and uveitis—making early diagnosis particularly challenging (3, 19, 20).

Ancillary imaging is essential for evaluating the full extent of ocular disease and the presence of systemic involvement (14). Among these modalities, ultrasonography is the most valuable examination (14) with 75.9% of cases showing typical features of choroidal thickening and localized hypoechoic extrascleral extension (ESE) (14) Although this combination highly suggests choroidal EMZL, similar ultrasound findings may occur in choroidal metastases (2). CT and MRI serve as supplementary diagnostic tools. MRI provides superior soft-tissue resolution compared to CT, making it the preferred neuroimaging modality for this disease (14). In addition, PET-CT plays an important role in assessing systemic involvement and the prognosis of the lesion. FA and OCT findings are nonspecific for choroidal lymphoma.

Despite these findings, clinical diagnosis of choroidal lymphoma remains challenging. Histopathological biopsy constitutes the diagnostic gold standard. Given that most choroidal lymphomas exhibit extraocular involvement (e.g., conjunctiva, orbit, sphenoid bone, lacrimal gland, or optic nerve) (3), the initial biopsy site should prioritize superficial lesions such as conjunctiva or Tenon’s capsule (21). When superficial sites are inaccessible, episcleral or scleral biopsies are alternatives (22). Transretinal choroidal biopsy or orbital biopsy, though more invasive, could also be helpful (22–24). In advanced cases, enucleation may be necessary for definitive diagnosis.

Once choroidal lymphoma is diagnosed, early treatment improves prognosis and survival (25). External-beam radiotherapy is the primary treatment for localized choroidal lymphoma. Chemotherapy and immunotherapy are reserved for patients with multifocal or systemic involvement (26, 27). Molecularly targeted anti-CD20 therapy has also gained prominence (22). Overall, choroidal EMZL has a more favorable clinical course than other lymphomas (14). Regardless of treatment modality, patients achieve good outcomes: 78.6% (22 cases) attained complete remission after a median follow-up of 30.3 months (range 7.8–180.2 months), with no lymphoma-related deaths reported.

This case also highlights a missed opportunity for vision-preserving therapy. Had a definitive histological diagnosis been established at the initial presentation, several well-tolerated and effective treatment options could have been considered for this elderly patient, potentially salvaging the eye. For localized ocular disease, low-dose ocular radiotherapy or intravitreal rituximab injections have shown efficacy with favorable safety profiles (28). For systemic control, single-agent rituximab (an anti-CD20 monoclonal antibody) or combination therapy with rituximab and lenalidomide are effective regimens for indolent lymphomas in the elderly (29, 30). This underscores the paramount importance of pursuing an early histological diagnosis in patients with suspicious choroidal masses to enable timely, sight-saving interventions.

## Conclusion

In conclusion, we present a rare case of primary choroidal lymphoma with extrascleral extension and bone marrow involvement. Due to its insidious onset and nonspecific manifestation, clinicians must be aware of the possibility of choroidal lymphoma in a patient presenting with choroidal masses, particularly when it is accompanied by hypoechoic peri-optic nerve extrascleral extension. Moreover, although primary choroidal EMZL is an indolent, low-grade tumor, systemic involvement can occur and should be actively investigated.

## Data Availability

The raw data supporting the conclusions of this article will be made available by the authors, without undue reservation.
