# Correction: Primary choroidal lymphoma with extrascleral extension and bone marrow involvement: a case report

**DOI:** 10.3389/fmed.2026.1794868

**Published:** 2026-02-16

**Authors:** Frontiers Production Office

**Affiliations:** Frontiers Media SA, Lausanne, Switzerland

**Keywords:** extranodal marginal zone B-cell lymphoma, choroid, lymphoma, bone marrow, involvement

The **Acknowledgements** statement was omitted. It should be as follows:

“The authors thank Editage (www.editage.cn) for English language editing.”

The Generative AI statement was erroneously given as:

“The authors declare that Gen AI was used in the creation of this manuscript. The authors thank Editage (www.editage.cn) for English language editing.

Any alternative text (alt text) provided alongside figures in this article has been generated by Frontiers with the support of artificial intelligence and reasonable efforts have been made to ensure accuracy, including review by the authors wherever possible. If you identify any issues, please contact us.”

The correct Generative AI statement is:

“The authors declare that no Gen AI was used in the creation of this manuscript.

Any alternative text (alt text) provided alongside figures in this article has been generated by Frontiers with the support of artificial intelligence and reasonable efforts have been made to ensure accuracy, including review by the authors wherever possible. If you identify any issues, please contact us.”

The original version of this article has been updated.

