# Supplementary figures and images for: Primary choroidal lymphoma with extrascleral extension and bone marrow involvement: a case report
Source: Front Med (Lausanne). 2025 Oct 27;12:1638453. doi: 10.3389/fmed.2025.1638453 (PMC12597990; doi:10.3389/fmed.2025.1638453)

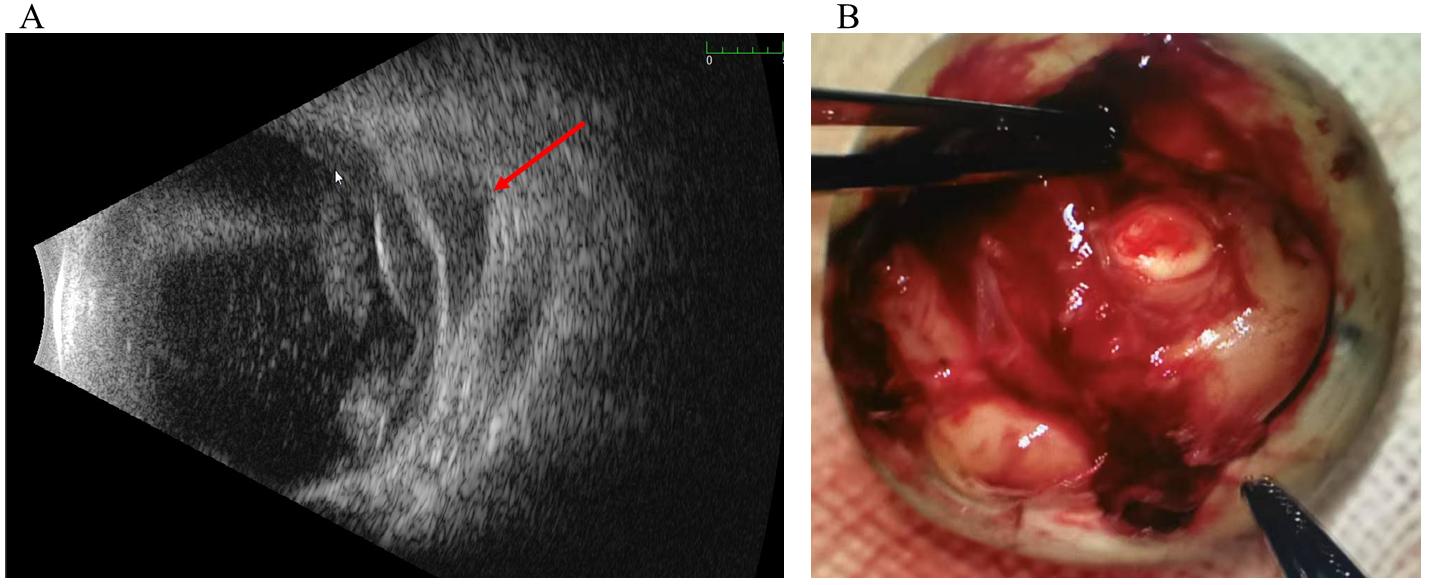

Supplement: Supplementary file 1 [file Image_1.JPEG]

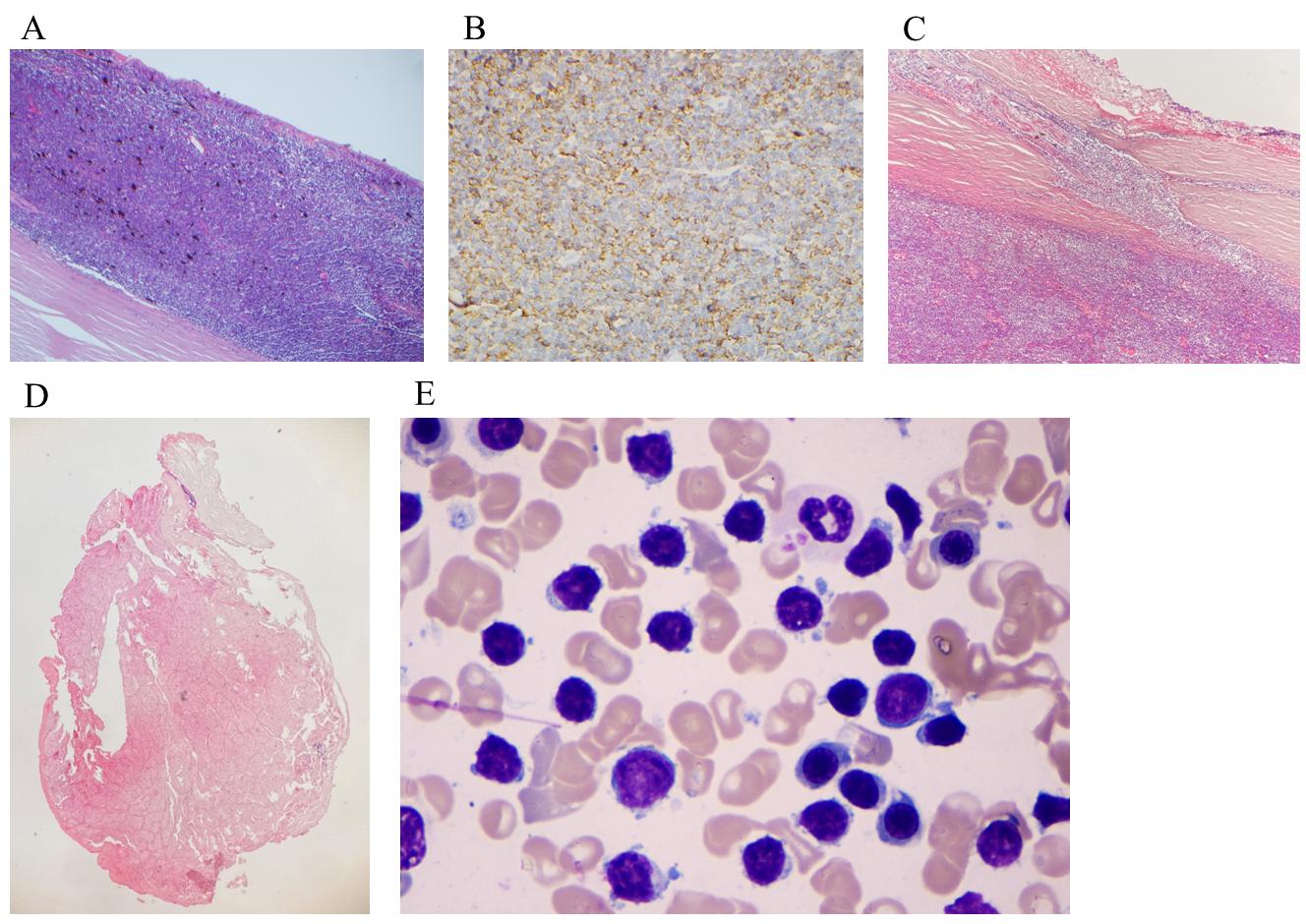

Supplement: Supplementary file 2 [file Image_2.JPEG]
